# p16, Cyclin D1, and HIF-1**α** Predict Outcomes of Patients with Oropharyngeal Squamous Cell Carcinoma Treated with Definitive Intensity-Modulated Radiation Therapy

**DOI:** 10.1155/2012/685951

**Published:** 2012-07-24

**Authors:** Asal S. Rahimi, David D. Wilson, Drew K. Saylor, Edward B. Stelow, Christopher Y. Thomas, James F. Reibel, Paul A. Levine, David C. Shonka, Mark J. Jameson, Paul W. Read

**Affiliations:** ^1^Department of Radiation Oncology, University of Texas Southwestern, 5323 Harry Hines Boulevard, Dallas, TX 75390, USA; ^2^Department of Radiation Oncology, University of Virginia, P.O. Box 800383, Charlottesville, VA 22908, USA; ^3^Department of Pathology, University of Virginia, P.O. Box 800383, Charlottesville, VA 22908, USA; ^4^Division of Hematology and Oncology, School of Medicine, Wake Forest University, Watlington 1, Medical Center Boulevard, Winston-Salem, NC 27157, USA; ^5^Department of Otolaryngology, University of Virginia, P.O. Box 800383, Charlottesville, VA 22908, USA

## Abstract

We evaluated a panel of 8 immunohistochemical biomarkers as predictors of clinical response to definitive intensity-modulated radiotherapy in patients with oropharyngeal squamous cell carcinoma (OPSCC). 106 patients with OPSCC were treated to a total dose of 66–70 Gy and retrospectively analyzed for locoregional control (LRC), disease-free survival (DFS), and overall survival (OS). All tumors had p16 immunohistochemical staining, and 101 tumors also had epidermal growth factor receptor (EGFR) staining. 53% of the patients had sufficient archived pathologic specimens for incorporation into a tissue microarray for immunohistochemical analysis for cyclophilin B, cyclin D1, p21, hypoxia-inducible factor-1**α** (HIF-1**α**), carbonic anhydrase, and major vault protein. Median followup was 27.2 months. 66% of the tumors were p16 positive, and 34% were p16 negative. On univariate analysis, the following correlations were statistically significant: p16 positive staining with higher LRC (*P* = 0.005) and longer DFS (*P* < 0.001); cyclin D1 positive staining with lower LRC (*P* = 0.033) and shorter DFS (*P* = 0.002); HIF-1**α** positive staining with shorter DFS (*P* = 0.039). On multivariate analysis, p16 was the only significant independent predictor of DFS (*P* = 0.023). After immunohistochemical examination of a panel of 8 biomarkers, our study could only verify p16 as an independent prognostic factor in OPSCC.

## 1. Introduction

The current treatment recommendations for patients with oropharyngeal squamous cell carcinoma (OPSCC) remain based largely on clinical parameters such as clinical stage and performance status with less emphasis on underlying tumor biology [1]. The current National Comprehensive Cancer Network (NCCN) treatment guidelines result in high cure rates for patients with OPSCC but are associated with significant acute toxicity, long-term morbidity, reduced functional status, and poor quality of life for many patients. In an effort to minimize toxicity, there is evidence that primary surgical therapy for T1-2 OPSCC can lead to good oncologic and functional results [2, 3]. Retrospective institutional studies have reported statistically significant associations of OPSCC outcomes with numerous tumor biomarkers linked to cell proliferation, growth factors, and hypoxia [4–18]. Prognostic molecular biomarkers have the potential to stratify OPSCC patients for clinical trials with the goal of appropriately targeting therapies and matching treatment intensity and toxicity with tumor sensitivity and curability.

Currently, the most well-established prognostic biomarker for OPSCC is the human papillomavirus (HPV). Recently reported subset analyses of Radiation Therapy Oncology Group (RTOG) 01-29 and Tran-Tasman Radiation Oncology Group (TROG) 02.02 have shown significantly improved outcomes of OPSCC patients with HPV-positive [6] and p16-positive (p16+) tumors, respectively [16]. We previously reported better clinical outcomes for patients with p16+ versus p16 negative (p16−) OPSCC treated with definitive intensity-modulated radiation therapy (IMRT) [8].

Because of this association with improved response to chemotherapy and radiotherapy, major cooperative groups are developing and enrolling patients into clinical trials for HPV-associated OPSCC. The presence of HPV (or the surrogate marker p16) is being used to select patients that are predicted to require less intensive therapy. While appropriate deintensification of therapy is a worthy goal, it is crucial to note that a subset of patients with HPV+ OPSCC may have poorer survival, while a group of patients with HPV− OPSCC may have better survival. Thus, additional biomarkers may be useful in conjunction with HPV/p16 to identify these subsets, which include patients that are at risk for failure in deintensification protocols.

Recent reports suggest that, particularly in the context of HPV-associated OPSCC, hypoxia may be a key regulator of response. Retrospective subset analyses of TROG 02.02 showed a trend for improved outcomes of patients with p16− tumors treated with cisplatin and the hypoxic cell sensitizer tirapazamine compared to cisplatin alone [16]. In addition, the Danish Head and Neck Cancer Group (DAHANCA 5) showed a trend for improved outcomes on retrospective subset analysis of patients with p16− tumors treated with the hypoxic cell sensitizer nimorazole [17]. Proteins that are expressed under hypoxic conditions, such as HIF-1*α*, may be predictors of response to radiotherapy and may be able to augment the prognostic value of HPV/p16 status. Similarly, other proteins correlated with outcome in head and neck squamous cell carcinoma (HNSCC) may be useful prognostic biomarkers in OPSCC.

In the present study, we report on eight immunohistochemical markers that have the potential to stratify patients with OPSCC. These markers have been previously reported to be prognostically significant for patients with HNSCC: p16, EGFR, cyclophilin B, cyclin D1, p21, HIF-1*α*, carbonic anhydrase, and major vault protein as described in Table 1. Our goal is to determine their clinical significance for patients with OPSCC treated with definitive IMRT and evaluate their potential for substratifying OPSCC patients beyond HPV/p16 status.

## 2. Methods and Materials

### 2.1. Clinical Treatment

Data were retrospectively collected on an Institutional Review Board-approved protocol for patients with histologically confirmed OPSCC treated with definitive IMRT at the University of Virginia between January 2002 and June 2010. Patients with prior head and neck cancer or irradiation, neck dissection before irradiation, or distant metastases at diagnosis were excluded, leaving 106 patients for analysis. Chemotherapy was indicated for patients with T3-4 primary tumors and/or N2-3 lymph node disease. Most chemotherapy regimens were platin-based, with a standard regimen of cisplatin 100 mg/m^2^ at days 1, 22, and 43 plus or minus a 5-FU agent. Some chemotherapy regimens also included a taxol or cetuximab. Neck dissections were performed on patients with bulky N2-3 nodal disease regardless of response to radiation, or on patients with concerning imaging 4–6 weeks after radiation completion. Patients were treated with conventionally fractionated radiation to 66–70 Gy to gross disease and 50 Gy to elective nodal volumes as previously reported [8].

### 2.2. Immunohistochemistry

57 patients analyzed in this study had sufficient archived formalin-fixed paraffin-embedded pretreatment primary tumor specimens to be included in a tissue microarray, which was constructed using three 0.6 mm cores of tumor per case. Immunohistochemistry was performed using a DAKO Autostainer. Primary antibodies included carbonic anhydrase (Abcam ab15086; titration: 1 : 1000; pressure retrieval), cyclin D1 (Epitomics 4202-1; titration: 1 : 75; pressure retrieval), cyclophilin B (Abcam ab 16045; titration: 1 : 400; pressure retrieval), EGFR (DAKO M3563; titration: 1 : 200; pressure retrieval), HIF-1*α* (Novus Biologicals NB100-131E3; titration: 1 : 1600; pressure retrieval), major vault protein (MVP) (Abnova H00009961-M01; titration: 1 : 1600; pressure retrieval), p21 (Santa Cruz SC-6246; titration: 1 : 100; pressure retrieval), and p16 (BD Biosciences 550834; titration: 1 : 100; pressure retrieval).

Immunohistochemical results were scored: p16 was considered “positive” if strong nuclear and cytoplasmic staining was present in more than 60% of tumor cells [9, 20]; p21 and cyclin D1 were scored semiquantitatively based upon percentage of nuclei staining (0 = 0%; 1 = 1–25%; 2 = 26–50%; 3 = 51–75%; 4 = 76–100%); EGFR was scored based upon percentage of cells with membranous staining (0 = 0%; 1 = 1–25%; 2 = 26–50%; 3 = 51–75%; 4 = 76–100%); cyclophilin B was scored based upon percentage of cells showing cytoplasmic staining (0 = 0%; 1 = 1–25%; 2 = 26–50%; 3 = 51–75%; 4 = 76–100%); MVP was scored based on percentage of cells with cytoplasmic staining and intensity of the staining (0 = 0%; 1 = 1–25%; 2 = 26–50%; 3 = 51–75%; 4 = 76–100% X 1 = mild; 2 = moderate; 3 = intense); HIF-1*α* was scored based on percentage of cells showing staining and intensity of staining (0 = 0%; 1 = 1–25%; 2 = 26–50%; 3 = 51–75%; 4 = 76–100% X 1 = mild; 2 = moderate; 3 = intense), with scores for both nuclear and cytoplasmic staining; carbonic anhydrase was scored based upon percentage of cells showing membranous and cytoplasmic staining and intensity of staining (0 = 0%; 1 = 1–25%; 2 = 26–50%; 3 = 51–75%; 4 = 76–100% X 1 = mild; 2 = moderate; 3 = intense). When semiquantitative variables were converted to binary variables for Kaplan-Meier survival analysis, any score greater than 0 was considered to be a positive upregulation of that biomarker.

### 2.3. Statistical Analysis

 LRC was defined using the time between the date of diagnosis and the date of first local or regional recurrence. Patients without evidence of local or regional recurrence were censored at date of last followup. DFS was defined using the time between the date of diagnosis and the date of first disease recurrence or death from any cause. Patients without any recurrence or death were censored at the date of last followup. OS was defined using the time between the date of diagnosis and the date of death from any cause.

*t*-tests, Fisher's exact tests, and chi-square tests were used to assess differences in patient characteristics. A *P* value threshold of 0.05 was used for statistical significance. SPSS statistical software (version 16.0.1 for Windows; SPSS Inc, Chicago, Illinois) and SAS statistical software (version 9.2 for Windows; SAS Inc, Cary, North Carolina) were used for statistical analysis. Univariate and multivariate analyses were conducted using the Cox proportional hazards regression model. The univariate analysis included the 8 biomarkers as well as age, gender, stage, tobacco use (≥5 pack-year history), regular daily alcohol use (>1 drink daily), and treatment with chemotherapy. Predictors that were found to have *P* values less than 0.05 on univariate analysis were included in the multivariate analysis model. The log-rank test was used to compare Kaplan-Meier survival curves. EGFR, HIF-1*α*, and cyclin D1 were transformed to binary variables for the Kaplan-Meier curves based on the presence or absence of upregulation of each biomarker. The Cochran-Armitage trend test was used to test the association between p16 status and cyclin D1 score.

## 3. Results and Discussion

### 3.1. Results

Patients with evaluable immunohistochemical data included 106 patients for p16, 101 for EGFR, 46 for cyclophilin B, 59 for cyclin D1, 60 for p21, 58 for HIF-1*α*, 57 for carbonic anhydrase, and 55 for major vault protein. The median followup for the entire cohort was 27.2 months (range 2.4–96.8 months). Patient demographics are reported in Table 2.

Results of univariate analysis of the 8 studied proteins are shown in Table 3. Chemotherapy use and age were significant predictors of DFS. p16, cyclin D1, and HIF-1*α* (nuclear) were significantly associated with DFS. p16 and cyclin D1 were also significantly associated with LRC, while HIF-1*α* (nuclear) and EGFR demonstrated a trend towards significance with LRC as an endpoint.

Variables with *P* values less than 0.05 in univariate analysis were included in the multivariate analysis model for DFS. Thus, the following prognostic variables were included in the multivariate model: age, chemotherapy use, p16, cyclin D1, and HIF-1*α* (nuclear). Tumors from 57 patients had sufficient data on the biomarkers of interest for inclusion in the multivariate analysis. On multivariate analysis, p16 was found to be the only predictor independently associated with DFS (Table 4). 

**Figure 1:**
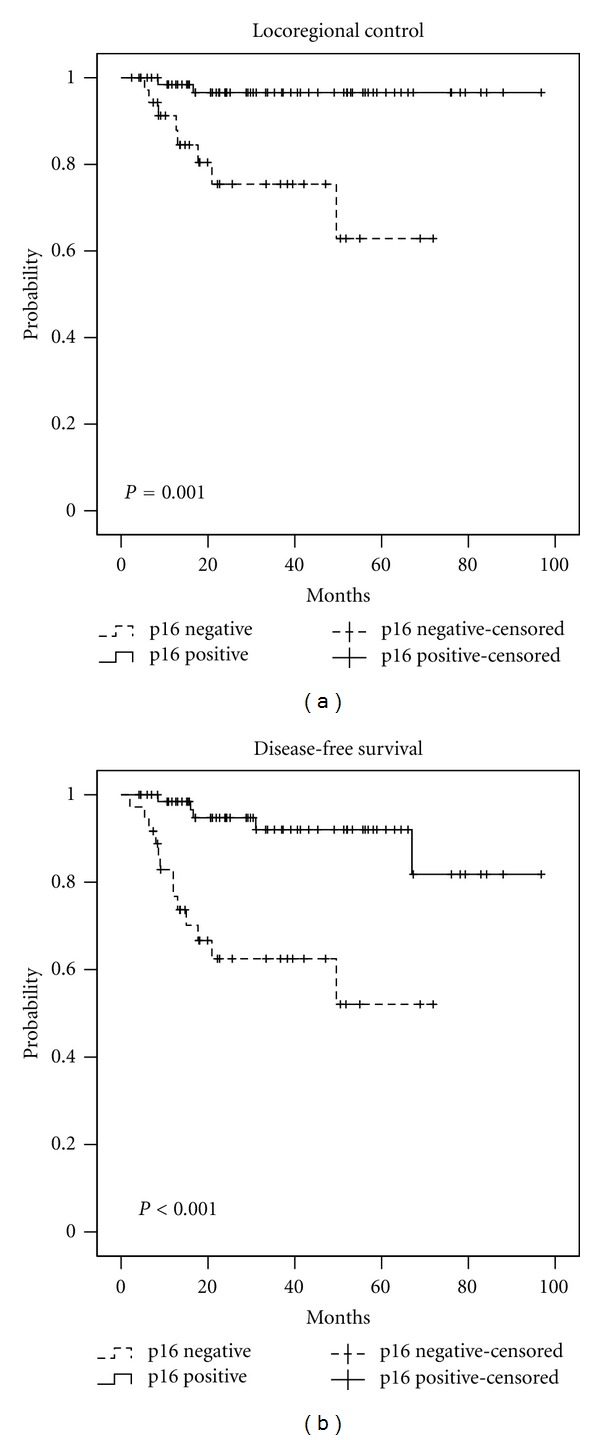
Locoregional control and disease-free survival stratified by p16 status.

**Figure 2:**
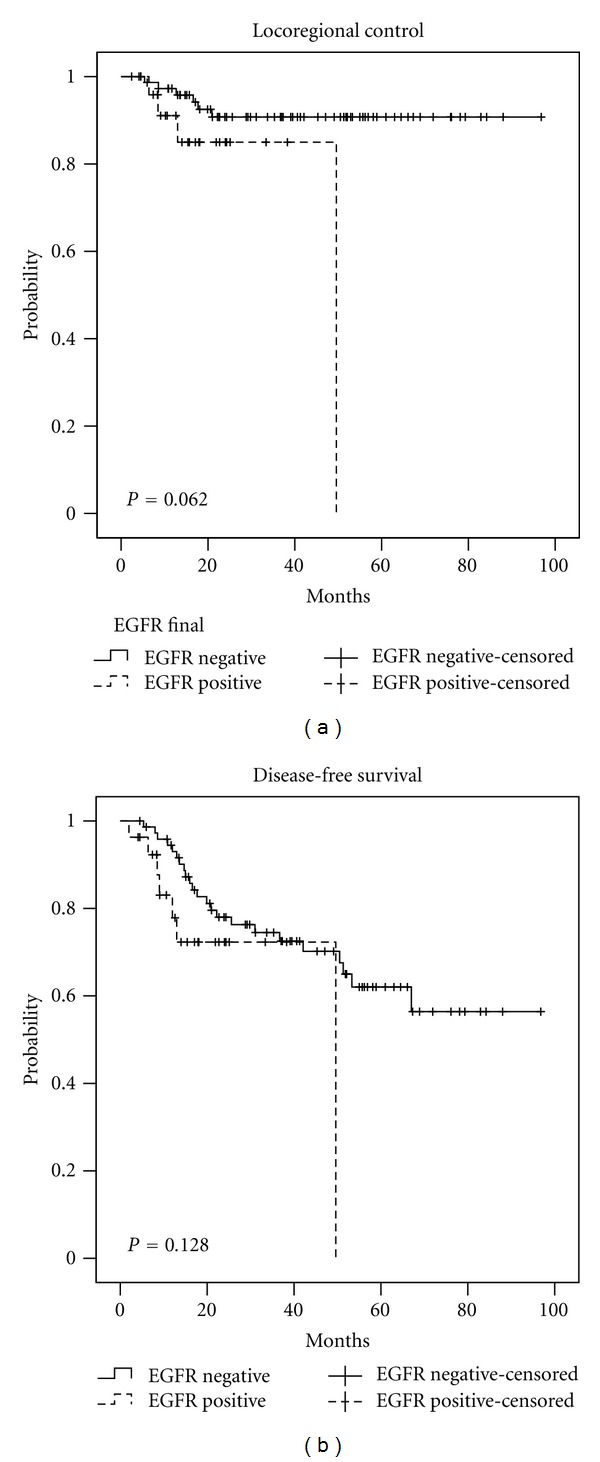
Locoregional control and disease-free survival stratified by EGFR status.

**Figure 3:**
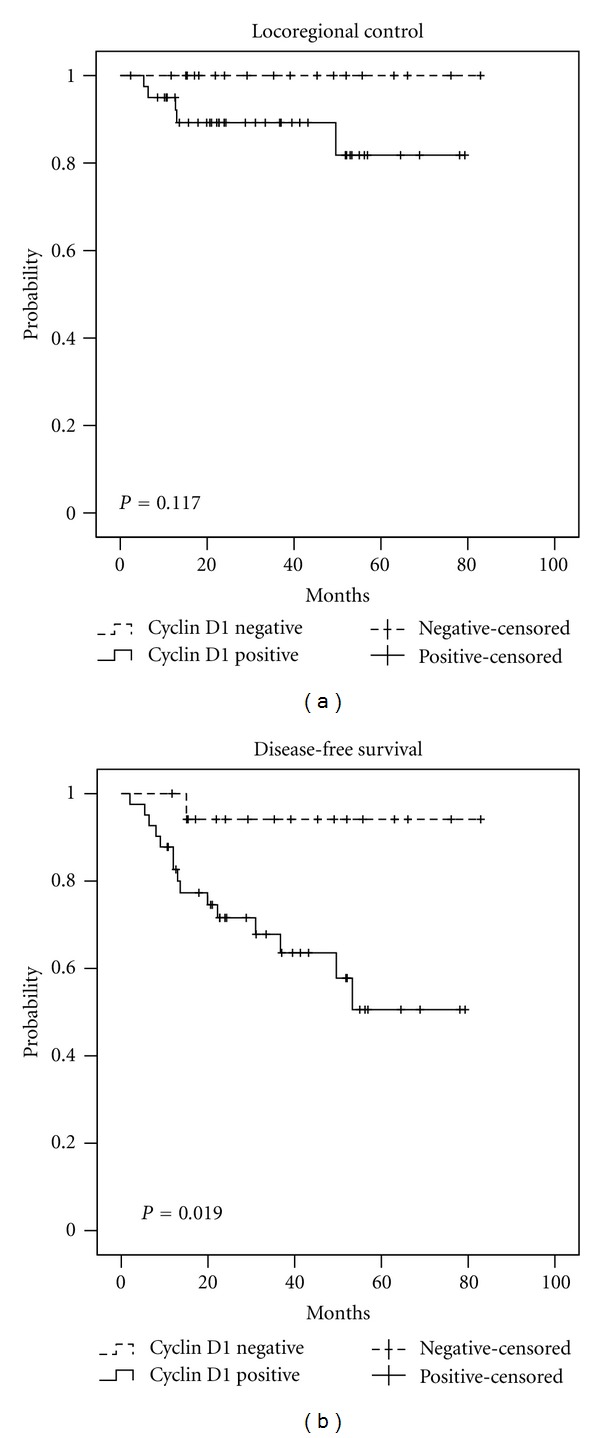
Locoregional control and disease-free survival stratified by cyclin D1 status.

**Figure 4:**
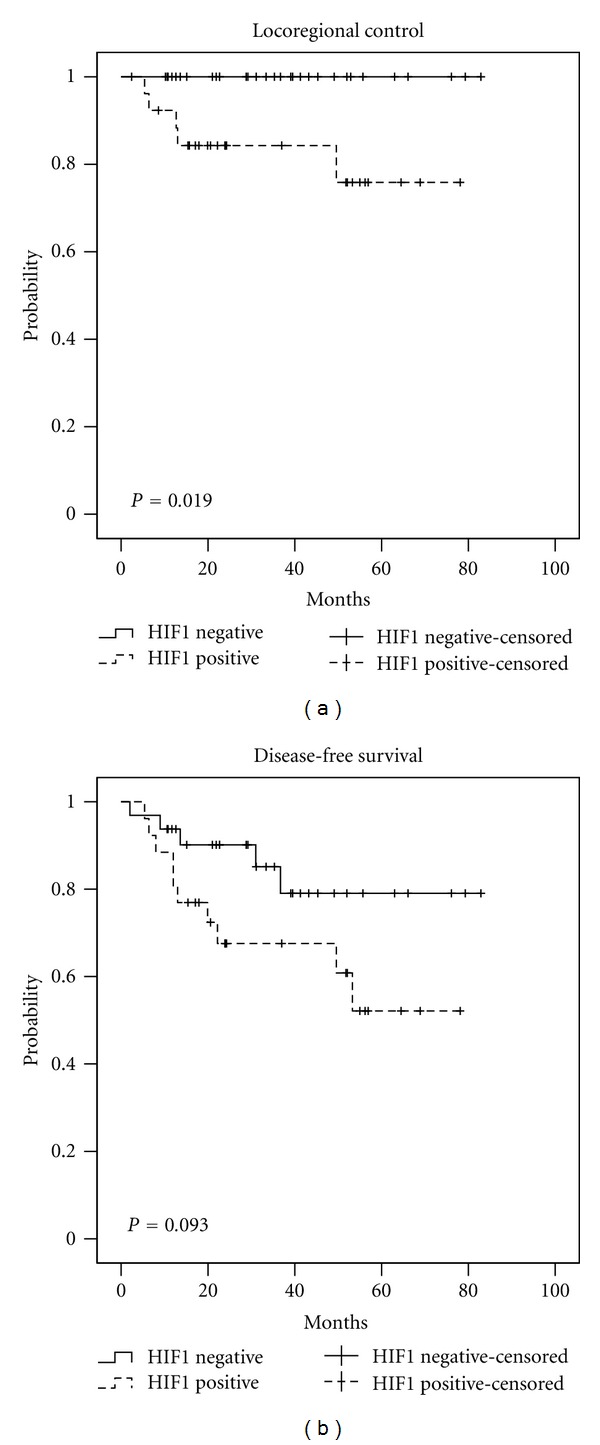
Locoregional control and disease-free survival stratified by HIF-1*α* (nuclear) status.

For the entire cohort, the 3-year OS was 77.7%, the 3-year LRC was 89.7%, and the 3-year DFS was 74.0%. The 3-year LRC, DFS, and OS stratified by upregulation for p16, EGFR, HIF-1*α* (nuclear) and cyclin D1 can be seen in Table 5. The Kaplan-Meier curves for LRC and DFS stratified by p16, EGFR, cyclin D1, and HIF-1*α* can be seen in Figures 1, 2, 3, and 4, respectively. 

As seen in Table 6, there was an inverse relationship between p16 status and cyclin D1 staining level. The Cochran-Armitage Trend test had *P* value of <0.001, confirming p16+ tumors are significantly associated with low cyclin D1 levels and p16− tumors associated with high cyclin D1 levels. 

### 3.2. Discussion

Investigators have reported the prognostic significance of multiple biomarkers on the outcomes of patients with HNSCC. Various studies have included patients with primary tumors from multiple head and neck cancer sub-sites and treated with various combinations of surgery, radiation, and/or chemoradiation to their primary tumors. p16 is a cyclin-dependent kinase inhibitor that has been closely correlated with HPV status in patients with OPSCC, and its expression or lack thereof was superior to all clinical or pathologic parameters in one analysis [9]. In a meta-analysis of 37 studies reporting on HPV status and outcome for HNSCC, patients with HPV+ OPSCC had a 28% reduced risk of death compared to patients with HPV− OPSCC [7]. The exact mechanism(s) of enhanced p16+ or HPV+ OPSCC radiation responsiveness remain unknown. One hypothesis is that p16+ tumors have higher proliferation rates leading to high rates of radiation-induced mitotic cell death [21], making p16+ tumors more sensitive to radiation, as a high fraction of the tumor cells would be in the radiosensitive G2/M part of the cell cycle when treated with daily fractionated radiotherapy.

We previously reported an approximate 25–30% improvement of outcomes for p16+ versus p16− OPSCC tumors at our institution. For patients with p16+ and p16− tumors, the 3-year locoregional progression-free survival rate was 97.8% and 73.5% (*P* = 0.006) and the DFS rate was 88.2% and 61.4%, respectively (*P* = 0.004) [8]. In this present study, we investigated the prognostic significance of multiple previously reported protein biomarkers in addition to p16 for patients with OPSCC treated with definitive IMRT in an attempt to further stratify patients with p16+ and p16− tumors into significantly prognostic subgroups. 

After performing univariate and multivariate analysis to determine the relative importance of the eight protein markers in the current study, we identified that p16 was the biomarker with the highest impact on DFS and LRC. However, on univariate analysis, cyclin D1 and HIF-1*α* also showed prognostic significance towards DFS. 

Cyclin D1 is a cell cycle regulator that participates at the G1-S cell cycle checkpoint, and previous authors have reported that p16+ tumors generally are cyclin D1- and vice versa [22]. The Cochran-Armitage Trend Test comparing p16 status to cyclin D1 levels in our patients confirmed that the two biomarkers were significantly inversely associated (*P* < 0.001). Since cyclin D1 is inversely associated with HPV/p16, it is unlikely to provide additional prognostic information beyond that given by p16. This was confirmed in our multivariate analysis which found that cyclin D1 had no prognostic significance for DFS independent of p16 status. Therefore, cyclin D1 did not give additional prognostic information for a patient whose p16 status is already known.

Reimers et al. reported that patients with EGFR overexpressing tumors had a 5-year DFS rate of 47% compared to 74% for patients with tumors without EGFR overexpression, although EGFR status was not statistically significant on univariate or multivariate analysis [9]. Similar to this study by Reimers et al., we did not find EGFR status to be statistically significant. Bonner et al. reported the 5-year results of a phase III trial showing improved outcomes for patients with HNSCC treated with concurrent cetuximab and radiation compared to radiation alone [23]. Interestingly, patients with EGFR+ in <50% of the cells had more clinical improvement than patients with EGFR+ in >50% of cells despite the fact that the study therapy was targeting the EGFR [23]. Therefore, although cetuximab targets the EGFR and benefits patients with HNSCC, we do not have clinical evidence that the subgroup that benefits the most is the group with the highest overexpression of EGFR. 

Hypoxia has been reported to promote genomic amplification and instability, driving increased metastatic potential, decreased proliferation, and chemotherapy resistance. HIF-1*α* has been studied extensively as an indirect assessment of tumor hypoxia, and investigators have shown increased tumor expression in patients with HNSCC to be independently associated with lower LRC, DFS and OS outcomes [4, 11]. Overgaard recently published a meta-analysis of hypoxia modification of radiotherapy in HNSCC in which he analyzed 4805 patients on 32 randomized trials and reported that hypoxia modified therapy improved locoregional control (OR, 0.71; 95% Cl, 0.63–0.80; *P* < 0.001) [24]. He concluded that this meta-analysis demonstrated that there is level 1a evidence in favor of adding hypoxic modification to radiotherapy of HNSCC. 

The TROG 02.02 phase III trial recently randomized patients with HNSCC to radiation with cisplatin ± tirapazamine. Subset analysis found a trend favoring the tirapazamine arm for improved LRC in patients with p16− tumors (HR, 0.33; 95% CI, 0.09–1.24; *P* = 0.13) with 2-year LRC rates being 92% on the cisplatin and tirapazamine arm and 81% on the cisplatin alone arm [16]. A recent reanalysis of the DAHANCA 5 trial with the hypoxic cell sensitizer nimorazole found improved LRC when this drug was added to radiotherapy, and the benefit was restricted to patients with p16− tumors [17]. Neither of these trials reported the HIF-1*α* status of the patient's tumors. 

Our data suggests that only 35% of p16+ tumors are hypoxic, and 65% of p16− tumors are hypoxic based on HIF-1*α* analysis. If p16− tumors are more likely to be hypoxic, this may explain the subset analysis of improved outcomes for only p16− patients with tirapazamine and nimorazole. Given the increased cytotoxicity of hypoxic cell sensitizers, future clinical trials studying their concurrent use with radiation may consider targeting patients based on p16 and HIF-1*α* status for randomization and/or as a component of the eligibility criteria. However, our data showed that HIF-1*α* was a significant factor on univariate analysis, but on multivariate analysis it did not reach significance. Thus, p16 status is a stronger prognostic indicator than HIF-1*α* in our oropharyngeal patient cohort. 

The major limitation of our study is the low patient numbers after sub-stratification of the 106 patients. After substratification, there were only 57 OPSCC tumor specimens with all four p16, EGFR, cyclin D1, and HIF-1*α* immunohistochemistry. Another weakness was the use of several different chemotherapy regimens over the eight years of study. Despite this, we were still able to show protein markers were statistically significant for clinical outcomes of OPSCC patients treated with definitive IMRT. 

Despite being the current main determinate of clinical treatment recommendations, clinical stage was not a statistically significant parameter on univariate analysis. This may be because most patients (88.0%) had stage III/IV disease. Stage I/II patients were generally treated with radiation alone while stage III/IV patients were generally treated with chemotherapy and radiation, so their outcomes are not directly comparable. p16 is a useful marker for future clinical trials for OPSCC patients. EGFR and hypoxia have available targeted drug therapies that have been used in clinical trials concurrently with radiation, and future national trials will target HPV/p16-associated tumors for treatment deintensification strategies. Clinical trials using biomarker-stratified approaches are the best way to test if targeted concurrent therapies benefit specific subsets of OPSCC patients. These approaches are required to change national treatment recommendations from ones based mainly on stage to ones based on a more personalized approach using specific biomarker profiles. We encourage rapid accrual to current national biomarker-stratified clinical trials that will likely lead to this treatment paradigm change. 

## 4. Conclusions 

After immunohistochemical examination of a panel of 8 biomarkers, our study could only verify p16 as an independent prognostic factor in OPSCC. p16 remains the most valuable biomarker for patients with OPSCC tumors and is therefore useful for prospectively stratifying patients for treatment on intensification or deintensification clinical trials. 

## Figures and Tables

**Table 1:** Description of biomarkers.

Molecular marker	Description
p16^INK4a^	Tumor suppressor protein that acts as a cyclin-dependent kinase inhibitor to regulate the cell cycle and is a demonstrated surrogate marker for infection with HPV [7]. Specifically, overexpression of p16 is considered a biomarker for inactivation of tumor suppressor retinoblastoma protein (pRb) by the HPV E7 oncoprotein. P16 itself is a strong independent positive prognostic indicator in OPSCC [7, 8], even when compared to reliable predictors of survival such as stage and grade [8].

Epithelial growth factor receptor (EGFR)	Transmembrane tyrosine kinase receptor that regulates cell growth in response to activation by growth factor ligands. Its overexpression is associated with increased tumor cell proliferation, angiogenesis, loss of differentiation, and reduced apoptosis [12]. Previous studies have shown that EGFR is significantly related to decreased overall survival in OPSCC [10].

Cyclophilin B	Has peptidyl-prolyl isomerase enzymatic activity that functions as a transcriptional inducer for Stat5 and as a ligand for CD147. It is thought to enhance tumorigenesis and motility through multiple mechanisms [18, 19].

Hypoxia inducible factor-1*α* (HIF-1*α*)	Transcription factor that responds to a decrease in oxygen levels within a cell and can be used as an indirect assessment of tumor hypoxia levels. Increased HIF-1*α* levels have been independently associated with decreased locoregional control, disease-free survival, and overall survival in OPSCC [11].

Major vault protein (MVP)	MVP is a protein that forms part of the ribonucleoprotein particle called vault, which has been implicated in the regulation of cellular signaling cascades and multidrug resistance [13, 14].

Carbonic anhydrase 9	Carbonic anhydrase 9 is an enzyme that is overexpressed in hypoxic tumor cells. High co-expression of carbonic anhydrase 9 and MVP has been associated with a poor probability of locoregional control in patients with head and neck squamous cell carcinoma [14].

p21	Cyclin-dependent kinase inhibitor that regulates the cell cycle at G1 and inhibits cell growth. p21 overexpression has been strongly associated with HPV16-positive tonsillar squamous cell carcinoma and is a favorable prognosticator [15].

Cyclin D1	Cell cycle regulator that participates at the G1-S portion of the cell cycle. HPV-positive tonsillar tumors have been shown to be associated with lower cyclin D levels [15].

**Table 2:** Patient demographics.

Overall	p16 negative	p16 positive	*P* value	
*n*	106	36	70	
Mean age (years)	58.1	59.2	57.5	0.461^∗^
Median followup (months)	27.2	26.9	37.2	
Gender				
Male	83 (78.3%)	25 (69.4%)	58 (82.9%)	0.113^†^
Female	23 (21.7%)	11 (30.6%)	12 (17.1%)	
Received chemotherapy (%)	65 (61.3%)	18 (50.0%)	47 (67.1%)	0.086^†^
Habits (%)				
Tobacco use	83 (78.3%)	35 (97.2%)	48 (68.6%)	0.000^††^
Alcohol use	68 (64.2%)	29 (80.6%)	39 (55.7%)	0.011^†^
Primary site (%)				0.002^††^
Tonsil	58 (54.7%)	20 (55.6%)	38 (54.3%)	
Base of tongue	41 (38.7%)	10 (27.8%)	31 (44.3%)	
Soft palate	6 (5.7%)	6 (16.7%)	0 (0.0%)	
Pharyngeal wall	1 (0.9%)	0 (0.0%)	1 (1.4%)	
Primary tumor classification				0.467^††^
T1	29 (27.4%)	9 (25.0%)	20 (28.6%)	
T2	52 (49.1%)	16 (44.4%)	36 (51.4%)	
T3	12 (11.3%)	4 (11.1%)	8 (11.4%)	
T4	13 (12.3%)	7 (19.4%)	6 (5.6%)	
Lymph node classification				0.393^††^
N0	14 (13.2%)	7 (19.4%)	7 (10.0%)	
N1	21 (19.8%)	8 (22.2%)	13 (18.6%)	
N2a	9 (8.5%)	1 (2.8%)	8 (11.4%)	
N2b	40 (37.7%)	15 (41.7%)	25 (35.7%)	
N2c	18 (17.0%)	4 (11.1%)	14 (20.0%)	
N3	4 (3.8%)	1 (2.8%)	3 (4.3%)	
Stage group				0.711^††^
I	0 (0.0%)	0 (0.0%)	0 (0.0%)	
II	10 (9.4%)	5 (13.9%)	5 (7.1%)	
III	23 (21.7%)	7 (19.4%)	16 (22.9%)	
IVA	69 (65.1%)	23 (63.9%)	46 (65.7%)	
IVB	4 (3.8%)	1 (2.8%)	3 (4.3%)	
IVC	0 (0.0%)	0 (0.0%)	0 (0.0%)	
Histology				0.007^††^
Well differentiated	5 (4.7%)	2 (5.6%)	3 (4.3%)	
Moderately differentiated	33 (31.1%)	15 (41.7%)	18 (25.7%)	
Poorly differentiated	29 (27.4%)	12 (33.3%)	17 (24.3%)	
Not otherwise specified	9 (8.5%)	5 (13.9%)	4 (5.7%)	
Basaloid	26 (24.5%)	2 (5.6%)	24 (34.3%)	
Lymphoepithelioma	1 (0.9%)	0 (0.0%)	1 (1.4%)	
Papillary	3 (2.8%)	0 (0.0%)	3 (4.3%)	

^∗^ *t*-test.

^†^Chi-square test.

^††^Fisher's exact test.

**Table 3:** Univariate Cox regression analysis.

Variable	Locoregional control			Disease-free survival		
	Hazard ratio	95% CI^∗^	*P* value	Hazard ratio	95% CI^∗^	*P* value
Age	0.992	0.934–1.054	0.796	1.054	1.018–1.090	**0.003**
Sex	0.497	0.063–3.950	0.509	1.831	0.809–4.147	0.147
Stage	1.004	0.384–2.622	0.994	0.851	0.518–1.398	0.524
Tobacco	32.13	0.073–14061	0.263	1.807	0.690–4.733	0.229
Alcohol	6.764	0.850–53.83	0.071	1.713	0.793–3.702	0.171
Chemotherapy	0.266	0.069–1.031	0.055	0.307	0.144–0.657	**0.002**
p16	0.105	0.022–0.497	**0.005**	0.155	0.070–0.341	**0.000**
Cyclin D1	2.368	1.071–5.237	**0.033**	1.831	1.247–2.688	**0.002**
HIF-1*α* (nuclear)	1.339	0.986–1.818	0.061	1.233	1.011–1.503	**0.039**
EGFR	3.236	0.880–11.90	0.077	1.971	0.809–4.798	0.135
Cyclophilin B	1.787	0.501–6.382	0.371	1.447	0.742–2.821	0.278
p21	1.679	0.751–3.754	0.206	1.235	0.802–1.901	0.338
HIF-1*α* (cytoplasmic)	0.910	0.601–1.377	0.655	0.941	0.743–1.192	0.615
Carbonic anhydrase (membranous)	1.075	0.888–1.303	0.458	0.991	0.869–1.130	0.890
Carbonic anhydrase (cytoplasmic)	0.659	0.307–1.413	0.284	0.969	0.763–1.231	0.797
Major vault protein	1.178	0.903–1.537	0.226	1.066	0.928–1.225	0.365

^∗^Confidence interval.

**Table 4:** Multivariate Cox regression analysis.

Variable	Disease-free survival		
	Hazard ratio	95% CI^∗^	*P* value
Age	1.032	0.981–1.085	0.222
Chemotherapy	0.649	0.195–2.164	0.482
p16	0.096	0.013–0.720	**0.023**
Cyclin D1	1.087	0.540–2.188	0.814
HIF-1*α* (nuclear)	1.212	0.945–1.555	0.130

^∗^Confidence interval.

**Table 5:** Summary of survival results stratified by biomarker status.

Markers	*n*	3-year LRC	3-year DFS	3-year OS
All Patients	106	89.7%	74.0%	77.7%

p16+	70	96.6%	88.9%	92.8%
p16−	36	75.4%	46.3%	49.8%
*P* value^§^	**0.001**	**<0.001**	**<0.001**

EGFR+	27	85.0%	72.3%	82.3%
EGFR−	74	90.7%	74.5%	76.6%
*P* value^§^	0.062	0.128	0.981

HIF-1*α* (nuclear)+	26	84.3%	67.6%	76.6%
HIF-1*α* (nuclear)−	32	100%	85.1%	90.1%
*P* value^§^	**0.019**	0.093	0.328

Cyclin D1+	41	89.2%	67.8%	78.2%
Cyclin D1−	18	100%	94.1%	92.9%
*P* value^§^	0.117	**0.019**	0.094

^§^Log-rank test.

**Table 6:** Tumor counts stratified by p16 and cyclin D1 staining.

		Cyclin D1 (% of cells staining)				
	*n*	0%	1–25%	26–50%	51–75%	76–100%
p16 negative	21	1	0	6	7	7
p16 positive	38	17	9	8	4	0

Cochran-Armitage Trend test: *P* < 0.001.
